# A multidimensional study of public satisfaction with the healthcare system: a mixed-method inquiry in Ghana

**DOI:** 10.1186/s12913-021-07288-1

**Published:** 2021-12-09

**Authors:** Padmore Adusei Amoah, Kingsley Atta Nyamekye, Ebenezer Owusu-Addo

**Affiliations:** 1 School of Graduate Studies, Institute of Policy Studies, Department of Applied Psychology, Lingnan University, 8 Castle Peak Rd., Tuen Mun, Hong Kong (SAR), China; 2 Department of Planning and Sustainability- School of Geo-Sciences, University of Energy and Natural Resources, Sunyani, Ghana; 3 Bureau of Integrated Rural Development, Kwame Nkrumah University of Science & Technology (KNUST), Private Mail Bag, University Post Office, Kumasi, Ghana

**Keywords:** Public satisfaction, Healthcare system, Social ecology, Social capital, Ghana

## Abstract

**Background:**

Many governments in sub-Saharan Africa have recently sought to improve their health systems by increasing investment in healthcare facilities and introducing social insurance programmes. However, little is known about the impact of these intended improvements on public perceptions about the healthcare systems. This article examines whether and why people of different socioeconomic and ideological backgrounds are satisfied (or not) with the current healthcare system in Ghana from a social ecological perspective.

**Method:**

Data were elicited from a cross-sectional mixed-method study conducted in four regions in Ghana in 2018. We used ordinal logistic regression and thematic analysis techniques to analyse the data.

**Results:**

Satisfaction with the healthcare system was generally low. From our quantitative study, intrapersonal factors (e.g., being older and having good health and well-being status); interpersonal factors (e.g., linking social capital); community factors (e.g., living in rural areas); and organisational and public policy factors (e.g., trust in the health system, favouring welfare policies, and being interested in politics) were positively associated with satisfaction with the healthcare system. These were corroborated by the qualitative study, which showed that poor attitudes of health personnel, financial constraints, perceived poor health facilities, and perceived inefficacy of services contribute to dissatisfaction with the healthcare system.

**Conclusion:**

Strategies to improve satisfaction with the healthcare system in Ghana should incorporate ecological perspectives by considering factors such as demographic profile, health needs, political orientation, issues of trust in the healthcare system, and the dynamics and impact of social relationships of populations concerned.

**Supplementary Information:**

The online version contains supplementary material available at 10.1186/s12913-021-07288-1.

## Background

Healthcare systems in many developing countries are fraught with a multiplicity of challenges that have over the years produced unsatisfactory outcomes [32, 41, 43, 55]. By healthcare system, we mean ‘the organisation and interrelation of the resources, be they human, physical and institutional that are directly implicated in healthcare’ ([32], p. 3). In Ghana, researchers have identified numerous deficiencies in the system, including rising unmet healthcare demands and increased out-of-pocket healthcare costs for users [32]. Saleh ([56], p. 3) adds that ‘although in many cases, quantity and access have increased, quality of care remains problematic’. Consequently, healthcare systems in Ghana and many sub-Saharan African countries have gradually been reinvented, particularly by detaching from colonial structures to accommodate modern demands and satisfy the current market-driven orientation [32]. Such policy innovations have cultivated new healthcare systems that are pluralistic and relatively more efficient [32, 56]. In Ghana, developments such as the establishment of social health insurance schemes (e.g., the National Health Insurance Scheme, NHIS), expansion of recruitment and training of health personnel, and the provision of modern healthcare facilities are common [7, 32]. Additionally, initiation of social welfare policies such as the Livelihood Empowerment against Poverty (LEAP) programme and free maternal healthcare policy are considered critical in protecting the health-related well-being of vulnerable groups in the country [23, 42, 50, 52, 53]. Key health indicators such as under-five mortality rate (a decline from 111 per 1000 live births in 2003 to 48 in 2018); maternal mortality (a decline of between 610 and 720 per 100,000 live births in 1990 to 308 in 2017); and increased life expectancy (from 57 in 2000 to 63 in 2016) are indicative of significant improvements in health outcomes in recent years [56, 61, 63].

However, a critical, yet unanswered question is whether the public is satisfied with the milestones achieved by the health system so far. This question is important because recent trends point to a changing demographic and epidemiological profile (e.g., rising non-communicable diseases alongside various infectious diseases) that are putting new demands on the healthcare system, which needs to be even more responsive [6, 12, 33, 34, 56]. Regrettably, most studies of healthcare-system-satisfaction globally have focused on patients, with a few on the public view [48]. This is also the situation in Ghana where existing research is dominated by the responses of health professionals and patients, perhaps due to under theorisation of views and perceptions of the public [2, 13, 49]. Moreover, empirical studies on public satisfaction with healthcare systems have predominantly been in high-income countries [36]. Thus, public opinion is not holistically understood and considered when shaping health services in developing and non-western countries [18, 36].

### Ghana’s health system: an overview

In line with Ghana’s decentralisation policy, which came into being in 1988, the health system of Ghana is highly decentralised. The Ministry of Health (MoH), together with sub-agencies such as Ghana Health Services (GHS) and the National Health Insurance Authority (NHIA), manage the health system of Ghana [32, 45, 56]. The MoH mainly deals with the policy and regulatory aspects of the system, while the GHS manages the delivery of different kinds of health services. GHS has the mandate to promote access to health services at the community, sub-district, district, and regional levels [51]. The NHIA oversees the National Health Insurance Scheme (NHIS)—a pro-poor financial buffer scheme—and other private health insurance schemes [32, 56].

Ghana’s health system is pluralistic in terms of the types of services offered. Both orthodox and traditional medical services are practised under various regulatory frameworks. The traditional medical services primarily comprise herbal remedies and spiritual healing [1]. Given its high patronage and importance in healthcare delivery, traditional medical practices were formalised in 2012 [1]. Notwithstanding, many householders still rely on unapproved herbal and spiritual care due to the poor integration of traditional medical services into the formal healthcare system [56]. The public sector contributes more than half of all healthcare expenditure on infrastructure, service provision, and training and recruitment of health personnel [32]. Private sector institutions, such as mission health services, provide significant supplementary and complimentary services [32].

With regard to human resources, Ghana is relatively well-serviced compared to countries with similar economic conditions [56, 60]. However, when benchmarked against international standards, the country is under-resourced. For instance, its average number of essential healthcare workers per 1000 population is 1.24 compared to the standard of 2.02 to 2.54 worldwide, and some 40% of these staff are non-clinical staff [56]. Notwithstanding, recent development plans have focused on improving ‘the health outcomes of the people’; by offering ‘financial protection’; and by ensuring ‘that the system is responsive, efficient, equitable, and sustainable’ ([56], p. 31).

Ghana’s healthcare system is now facing a double burden of disease with the rapid rise in non-communicable diseases (e.gs., hypertension and diabetes) alongside prevalent communicable diseases such as malaria [10, 33]. One of the key challenges facing the health system in Ghana is equity—ensuring that people in the same circumstances are treated the same [30]. Inequity in access to healthcare in Ghana arises from a multiplicity of factors, including the uneven rural-urban distribution of health resources, poverty, gender, and geographical location [26, 38].

### Aim of the study

This study adopts a social ecological lens, which is explained below, to explore how individual characteristics and their perceptions and ideological convictions, relating to various meso- and macro-level factors, affect the extent to which the public is satisfied or dissatisfied with the healthcare system in Ghana. Although research in this area is still evolving, examining public satisfaction with a healthcare system is considered one of the critical avenues for evaluating its performance and offering alternatives for system improvement [59]. After all, healthcare and health policy are meant to promote the well-being of people, and thus, their views, experiences, and expectations are critical to sustaining the system [36]. Satisfaction with the healthcare system in this study refers to whether the ordinary person is content with the overall characteristics of extant services, policies, institutions, personnel, facilities, and all factors related to public healthcare delivery. In contrast, the discontent of the ordinary person is counted as dissatisfaction with the healthcare system [27, 59].

### Conceptual framework and literature review

**Fig. 1:**
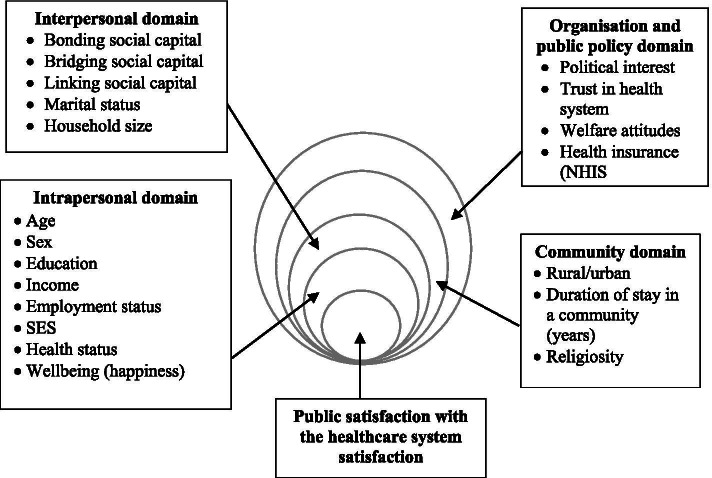
Conceptual model of the study based on the social ecological model

The study uses the social ecological model to offer a broader understanding of the correlates and perceptions of public satisfaction with the healthcare system [44]. The social ecological model posits that individuals are embedded in five multi-level domains that interactively shape the conditions of their lives as well as their expectations, standards, values, attitudes, behaviours and perceptions of their relationships with their environments [31, 57]. These multi-level domains comprise *intrapersonal, interpersonal, institutional or organisation, community characteristics, and public policy environment* domains [44]. Individual expectations and standards are inextricably connected with prevailing personal and ecological factors regarding satisfaction with the healthcare system [41]. In the context of this study, the five multi-level domains of the model are conceptualised into four (see Fig. 1), which are presented below relative to the phenomenon of public satisfaction with the healthcare system. We combined the domains on organisation and public policy domains because they concern institutional arrangements and policies that shape healthcare delivery.

The intrapersonal domain relates to existing characteristics such as age, sex, skills, knowledge, attitude, values and standards, and other demographic factors that predispose individuals to perceive a phenomenon positively or negatively, including healthcare systems [14, 28, 59]. For example, more educated persons (e.g. university graduates) are less likely to be satisfied with a health system than the less educated, according to research [19]. However, the rating of satisfaction with a health system also depends on individual needs or self-interest [36]. Andersen et al. [14] argues that these needs relate to the state of a person’s health and well-being which fundamentally determine how and whether they engage with the healthcare system [19]. People with poor health and those who are disconsolate are less likely to be content with the health system compared to those in good health due to outcome-based perception of the quality of services offered [2, 13, 48, 59].

According to the social ecological framework, individual perceptions are influenced by characteristics of the social environment in which they occur i.e., interpersonal domain. Factors such as social networks, social support, and quality of social relationships are essential to how people rate a phenomenon. People tend to evaluate their satisfaction with the healthcare system by comparing it with others in their social networks to ensure that their opinions are ‘correct’ [41]. In the literature on social capital (SC)—the social resources that are embedded in different kinds of social relationships—the role of social relationships in providing sufficient health services and thereby determining satisfaction with the health system is conceptualised as a social influence [9, 35, 41]. The interpersonal domain also connects to the perception of receptiveness of service providers. Attitudes of health personnel—particularly physicians and nurses—can influence the degree to which people are satisfied with the health services available to them [2, 55]. Poor attitudes of health personnel can adversely affect public satisfaction, but little is known about the actual extent of the problem in Ghana [2, 3].

The *community domain* in this study refers to a geographical and cognitive sense entity. It thus comprises factors such as whether a person lives in a rural or urban community, the duration of living in that community (which determines the physical and psychological bonds they form), cultural norms, and religious beliefs and groups. For instance, a study in mainland China found that rural residents tend to be more satisfied with the health care system than urban residents due to less pressure on facilities and services [64].

The *organisational and public policy domain*, as adapted for this study, focuses on ideational positions, attitudes, and perceptions on public policy and institutional arrangements aimed at improving the healthcare system, promoting health and well-being, and ‘policies that allocate programmatic resources’ such as welfare interventions and health insurance programmes ([44], p. 365). The effectiveness of public policies and institutional arrangements depend on how they are aligned with community characteristics, people’s expectations, and interpersonal facets of a community [44]. In view of these, factors such as trust, political perspectives, and welfare attitudes (e.gs., possession of egalitarian ideology) can affect satisfaction with a healthcare system, although these factors are rarely examined in extant research [27, 36, 59]. For instance, satisfaction with the health system is higher in places with functioning social insurance schemes where out-of-pocket payments for health services are low [20, 48, 62]. Bhatia et al. [18] argue that people who share similar ideological orientation as a given phenomenon or policy are likely to possess favourable opinions about the policies [18, 27, 48, 59].

## Methods

### Study design

This article emerged from a broader study of multidimensional aspects of well-being in Ghana, which was conducted from July to August 2018. The study employed a convergent mixed-method design using a cross-sectional approach to provide in-depth analyses of factors associated with public satisfaction with the healthcare system. Thus, qualitative and quantitative data were collected simultaneously and analysed separately, and the results were interpreted/discussed together [22].

The quantitative aspect examined the association of micro and macro factors with public satisfaction with the health system, as shown in Fig. 1. The qualitative aspect relied on an inductive approach to explore the nuances of the public’s perception of the healthcare system and the reasons for their satisfaction or dissatisfaction. We only gathered data from adults (18 years old and above) as these people have a higher chance of independently engaging with the healthcare system [3].

We used a multi-stage cluster sampling approach to gather data from four regions in the then ten administrative regions of Ghana in the quantitative part of the study to ensure a balanced sample. We purposively selected the four regions, but we selected 23 districts altogether from the regions using a simple random technique. The regions and the districts included Ashanti Region (eight districts), Upper East Region (five districts), Greater Accra Region (six districts), and Eastern Region (four districts). We chose these regions to ensure that the profile fairly represented the ethnic, religious, socioeconomic, and geographic characteristics of the country. The number of districts in each region was conveniently determined, but we ensured a fair representation of the selected regions and districts by allocating questionnaires proportionate to their population sizes. We derived the sample from the 36 rural and 70 urban communities that were selected using convenient and purposive sampling techniques to have a good balance of demographic, socioeconomic, and geographic characteristics. A minimum of 384 responses would have sufficed for all the regions, given the millions of people in the sampling frame [46]. However, we surveyed an adequate sample of 1381 individuals across the regions as non-probability sampling techniques mainly were applied. We targeted 384 responses for each region, although we did not meet this target in every region. Based on previous research and field experience, we used a systematic sampling approach to select one person from every fifth and second house in urban and rural areas, respectively, to interview [4, 34]. A face-to-face approach was predominantly used to interview participants, as many of them were illiterates, particularly among rural residents and older persons. The face-to-face approach also ensured that questions were less likely to be misinterpreted by participants. The questionnaire was in English, but trained interviewers administered them in the native dialect of participants.

In the qualitative study, we used the interpretivist paradigm to ensure that the views of participants were at the centre of data collection, analyses, and the interpretation of findings. This paradigm provides an opportunity to explore a research question from the perspective of people who have experienced the situation [22]. A semi-structured in-depth interview approach was used to gather data from 39 participants. Preliminary analysis was done after each interview, and by the 20th interview, data saturation was reached for almost all aspects of the study, but we carried out more interviews to ensure a balanced sample in terms of the characteristics of participants [21]. About 12 of these participants also took part in the quantitative study, and they were purposively selected as with all other participants. We used a maximum variation purposive sampling technique to select participants from Ashanti, Brong Ahafo, Upper East, and Greater Accra regions based on age, sex, educational attainment, and rural-urban residence, as shown in Table 3. The interview guides were pre-tested, and corrections were made based on the test. The interviews were conducted in-person at the homes and workplaces of participants by the first and second authors with support from (trained) research assistants who were fluent in the local languages of participants. Contents of the interviews included how people felt about the public healthcare system, the challenges they faced in accessing the health services, and the positive aspects of the system (Additional file 1: Appendix I shows the complete interview guide). Each interview lasted approximately 45 min, which were audio-recorded. Further details of the methods employed in this study have been reported elsewhere [7] and in Additional file 1: Appendix II. The Council for Scientific and Industrial Research (CSIR) (RPN 006/CSIR-IRB/2018) and Lingnan University Research Ethics Committee (EC-052/1718) approved the research protocol.

### Measures (quantitative study)

#### Dependent variable

The dependent variable was satisfaction with the health system. We asked this question: ‘In general, how satisfied are you with the healthcare system in Ghana?’ Our trained interviewers explained to participants that their concerns could be about the quality of care, cost of services, nature of facilities and infrastructure, health personnel, and any other issue about the healthcare system. This question was mainly about public healthcare services. Participants answered on a seven-point Likert scale ranging from: completely dissatisfied, very dissatisfied, fairly dissatisfied, neither satisfied nor dissatisfied, fairly satisfied, very satisfied, and completely satisfied. This approach is commonly used to measure public satisfaction with health systems [27].

#### Independent variables

The independent variables were categorised under the four domains of the social ecological model. The specific factors under each domain are shown in Fig. 1.

*Intrapersonal domain* included age (years), sex (male/female), educational attainment, monthly income/stipend, employment status, and self-rated socioeconomic status (SES, rated from 1, low to 10, high). We also measured health-related well-being in two ways. First, participants rated the state of their health (all things considered) in the past 4 weeks, and they responded on this scale: poor, fair, good, very good, and excellent [47]. Second, we asked participants to rate the extent of their happiness considering their lives in general, and they selected one of these responses: completely unhappy, very unhappy, fairly unhappy, neither happy nor unhappy, fairly happy, very happy, completely happy (see [25]). Table 1 shows details of the variables used.

**Table 1:** Descriptive statistics of variables in the study^a^

Variable	Frequency/Mean (SD) (***n*** = 1381)	Percentage
**Age**		
18–35	755	54.7%
36–49	217	15.7%
50+	409	29.6%
*Age (18–85 years) /Mean (SD)*	38.27 (16.05)	
**Sex**		
Male	748	54.2%
Female	633	45.8%
**Region of residence**		
Ashanti	546	39.5%
Greater Accra	203	14.7%
Eastern Region	206	14.9%
Upper East	426	30.8%
**Locality**		
Urban	813	58.9%
Rural	568	41.1%
**Duration of stay (in years)**		
Mean/SD	19.24/17.55	
Minimum-Maximum	0–85	
**Educational attainment**		
Never been to school	212	15.4%
Primary school	194	14.0%
JHS	344	24.9%
MSLC	102	7.4%
O′ Level	41	3.0%
A’ Level	24	1.7%
SHS/Vocational/Technical	243	17.6%
Tertiary	188	13.6%
Postgraduate	27	2.0%
**Employment status**		
Full-time employee	267	19.3%
Part-time employee	89	6.4%
Self-employed	438	31.7%
Pension/retired	81	5.9%
Student	199	14.4%
Housewife	33	2.4%
Unemployed	270	19.6%
**Religiosity**		
Extremely non-religious	27	2.0%
Very non-religious	20	1.4%
Somewhat non-religious	30	2.2%
Neither religious nor non-religious	103	7.5%
Somewhat religious	174	12.6%
Very religious	787	57.0%
Extremely religious	228	16.5%
**Monthly Income** (if employed) (Range: GH¢20–2500, ~ US$4–502.50)		GH¢480.73 (446.91) US$96.63 (89.83)
Low (US$ 0–57)	256	32.4%
Lower-middle (US$58–96)	162	20.5%
Middle (US$ 97–166)	89	11.3%
Upper-middle (US$ 167+)	118	14.9%
**Socioeconomic Status**		
Low (score 1–4)	311	39.3%
Middle (score 5–7)	405	51.2%
High (8–10)	71	9.0%
*Mean/SD*	*4.72(2.24)*	
**Household size**		
*Mean (SD)*	5.69 (3.11)	
**Marital status**		
Married	629	45.5%
Divorced	34	2.5%
Widowed	79	5.7%
Separated	33	2.4%
Living together as married	30	2.2%
Single	540	39.1%
**Trust in the healthcare system**		
Not at all	198	14.3
Not very much	331	24.0
To some extent	622	45.0
A great deal	230	16.7
**NHIS Subscription**		
Yes	640	46.3%
No	739	53.5%
**Health status**		
Poor	118	8.5
Fair	191	13.8
Good	495	35.8
Very good	339	24.5
Excellent	214	15.5
**Happiness**		
Completely unhappy	47	3.4
Very unhappy	67	4.9
Fairly unhappy	65	4.7
Neither happy nor unhappy	86	6.2
Fairly happy	382	27.7
Very happy	505	36.6
Completely happy	181	13.1
**Bonding SC**		
*Mean/SD*	*2.98(1.56)*	
*Minimum-Maximum*	*1–6*	
**Bridging SC**		
*Mean/SD*	*2.39(1.75)*	
*Minimum-Maximum*	*0–6*	
**Linking SC**		
*Mean/SD*	*2.33(2.02)*	
*Minimum-Maximum*	*0–7*	
**Welfare attitudes**		
Strongly disagree	92	6.7
Disagree	225	16.3
Neither agree nor disagree	241	17.4
Agree	574	41.6
Strongly agree	249	18.0
**Interest in politics**		
Not at all interested	383	27.7
Not very interested	229	16.6
Somewhat interested	276	20.0
Fairly interested	213	15.4
Very interested	279	20.2

^a^Some percentages may not sum up to 100 because of missing data

We measured the *interpersonal domain* using three proxies of social capital (SC), marital status, and household size. The three SC proxies included bonding SC (relationships between close friends and family), bridging SC (the weak relationship between a friend of a friend and distant neighbours), and linking SC (relationships with people and institutions and people of different socioeconomic and power statuses) [11]. We asked participants whether they had received any support (information, emotional and instrumental) from any person in each of the SC categories. The results were summed to establish a score for each SC category (see [6, 35]). Household size was an open-ended question, and respondents could state the number of people they shared daily living arrangements with. Marital status comprised six options, as shown in Table 1.

The *community domain* considered in this study included whether a person lived in a rural or urban area; the person’s religiosity rating (on a seven-point Likert scale); and the duration of his or her residence in a community, which was measured in years. Finally, the *organisation and public policy domain* was measured in four ways. First, we asked participants how much they trusted the public healthcare system, all things considered (responses: not at all, not very much, to some extent, and a great deal). Second, we asked them to rate how interested they were in politics, which was rated on a four-point Likert scale (Not very interested to very interested). Third, we measured attitudes towards welfare policies by eliciting responses to this statement: ‘the government should spend more money on welfare benefits for the poor, even if it leads to higher taxes’. Response options included strongly disagree, disagree, neither agree nor disagree, agree and strongly agree [37]. Fourth, we inquired whether participants had subscribed to the publicly available mutual health insurance programme, the NHIS. Table 1 contains more information about how each of the factors in Fig. 1 was measured.

### Data analyses

We employed the ‘side-by-side’ technique to analyse the data. This technique allows for qualitative and quantitative findings to be presented separately but discussed together through comparative analyses, which focus on differences and consistencies in the findings [22]. The quantitative data analysis began with descriptive statistics for all variables involved in the study, as shown in Table 1. We used an ordinal logistic regression technique to ascertain the factors associated with public satisfaction with the healthcare system. One model was constructed, and it included only variables that correlated significantly with the dependent variable in an earlier Spearman’s correlation analysis (Additional file 1: Appendix III) because the dependent variable was not normally distributed (Shapiro–Wilk test, W = .915, *p* = 0.001). There were few cases with missing values, and these values were usually replaced with the mean of the variables. We used SPSS version 26 to analyse the data. Significance levels for all associations were set at *p* < 0.05.

We employed a thematic analysis technique to analyse the qualitative data using NVivo software. The process was primarily inductive as the findings were dictated by the data instead of our conceptual model. This approach is consistent with the interpretivist paradigm we adopted as it ensures that the findings are dependent on the experiences and views of participants. Nonetheless, the identified themes were connected to the conceptual model in the presentation and interpretation of the findings. Two authors independently developed a codebook and coded the data through an open-coding method [21]. Each of them developed initial themes from the related codes. Both authors later compared their coding and initial themes iteratively to arrive at the final themes. We then submitted our analysis for an independent review by a scholar well-versed in qualitative research and familiar with the health system understudy to help validate our findings and interpretations.

## Findings

### Findings from the quantitative study

**Fig. 2:**
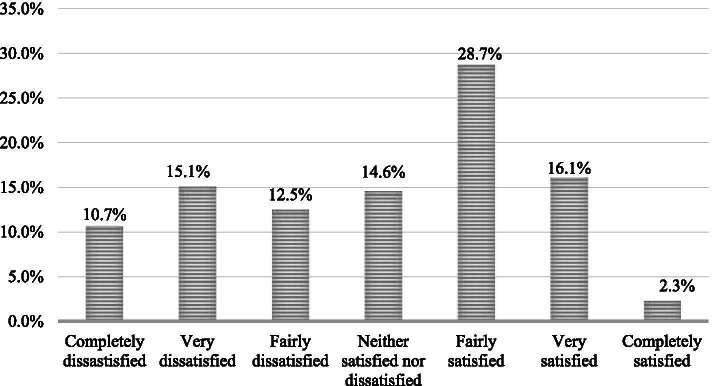
Public satisfaction with the healthcare system in Ghana

As shown in Table 1, the participants in the quantitative study had an average age of 38 years, with most of them being males. Most of the participants were interested in political issues and agreed that the government should spend more on welfare. A significant proportion of the participants had subscribed to the NHIS. Approximately half of them perceived their health status as either very good or excellent, while many of them were very content or completely content with their lives. In terms of SC, bonding SC was the commonest, followed by bridging and then linking SC. Finally, as shown in Fig. 2, responses to the question on satisfaction with the health system showed that less than half (47.1%) of the participants felt somewhat satisfied with the health system, 38.3% of participants were not satisfied with the health system while 14.6% of them were neither satisfied nor dissatisfied.

Factors associated with public satisfaction with the healthcare system are shown in Table 2. According to the odds ratios in Table 2, factors in the *intrapersonal domain,* older persons were 1.4% more likely to be satisfied with the health system than younger persons. Full-time employees and self-employed were 43 and 62%, respectively, more likely to be satisfied with the healthcare system than the unemployed. Satisfaction with the healthcare system was 16.8% higher among healthy persons and 9.1% higher among those who felt happy. Also, *interpersonal domain* factors such as people with linking SC were 19% more likely to describe the healthcare system in satisfactory terms. As regards the *community domain,* urban dwellers were 22.3% less likely than rural residents to be satisfied with the health system. On the *organisational and public policy domain,* participants with positive attitudes towards welfare and those with high interest in politics were 11.7 and 10.4% more likely to be satisfied with the health system, respectively. Lastly, those who trusted the health system were 73% more likely to be satisfied with it.

**Table 2:** Analysis of factors associated with satisfaction with the healthcare system by ordinal logistic regression

		Estimate (B)	Std. Error	***P***-value	Odds Ratio	95% Confidence Interval of B	
						Lower Bound	Upper Bound
1	**Intrapersonal domain**						
	Age	0.014	0.005	**0.004**	1.014	0.005	0.023
	Employment status						
	Full-time employee	0.358	0.170	**0.035**	1.430	0.025	0.691
	Part-time employee	−0.294	0.241	0.222	0.745	− 0.766	0.178
	Self-employed	0.487	0.153	**0.001**	1.627	0.187	0.786
	Pension/retired	0.427	0.280	0.128	1.533	−0.123	0.977
	Student	0.161	0.192	0.401	1.175	−0.215	0.537
	Housewife	−0.055	0.354	0.878	0.946	−0.749	0.640
	Unemployed (ref)						
	Self-perceived health status	0.155	0.053	**0.004**	1.168	0.051	0.259
	Happiness	0.087	0.040	**0.029**	1.091	0.009	0.165
2	**Interpersonal domain**						
	Bridging SC	0.008	0.005	0.144	1.008	−0.003	0.018
	Linking SC	0.174	0.057	**0.009**	1.190	0.048	0.283
3	**Community domain**						
	Area of residence						
	Urban	−0.252	0.117	**0.031**	0.777	−0.480	−0.023
	Rural (ref)						
	Duration of stay in current community	−2.496E-05	0.004	0.995	0.999	−0.008	0.008
4	**Organisational and public policy domain**						
	Welfare attitudes	0.111	.046	**0.032**	1.117	0.021	0.201
	Interest in politics	0.099	.038	**0.009**	1.104	.024	.173
	Trust in health system	0.548	.061	**0.000**	1.730	.429	.668
*Nagelkerke R-square*		0.171					

Odds ratios were computed using the resource by De Coster and Iselin [24]

### Findings from the qualitative study

Participants in the qualitative study were generally youthful, as most of them were between the ages of 18 and 35 years. There were more males than females, and the majority of them had attained Junior High School education, as shown in Table 3.

**Table 3:** Characteristics of participants in the qualitative study

Characteristic	Frequency ***N*** = 39	Percentage
**Age**		
18–35	17	43.6
36–49	12	30.8
50+	10	25.6
**Sex**		
Male	21	53.8
Female	18	46.1
**Educational attainment**		
Never being to school	3	7.69
Primary school	10	25.64
Junior High School/MLSC	17	43.59
SHS/A’Level/O’Level	5	12.82
Tertiary (including postgraduate)	4	10.26
**Regions**		
Ashanti	18	46.2
Brong Ahafo	9	23.1
Greater Accra	5	12.8
Upper East Region	7	17.9
**Locality**		
Urban	23	59.0
Rural	16	41.0

We identified *four non-exclusive* themes demonstrating the public’s satisfaction with the healthcare system. These themes were in various ways connected to the four domains of the social ecological model below. The themes included: attitudes and behaviours of health personnel; financial barriers to health care; poor and inadequate health facilities and equipment; and perceived efficacy of services and care. The dominant impression was that the healthcare system was performing poorly. Dissatisfaction with the health system often stemmed from issues relating to access to healthcare. However, there were a few participants who saw some positive sides to the health system. These, however, came with several reservations:

*It [the healthcare system] is good in some ways and bad in another way. Even the way they receive clients when you go to some places is bad* (Interview 6)

*It has two ways, neither good nor bad; the good outweighs the bad. A lot of things could be done to enable the doctors to serve patients better* (Interview 10).

### Attitudes and behaviours of health personnel

The first theme was related to the interpersonal domain of the social ecological model as it espoused the significance of attitudes of health personal towards potential users and their perception of the professional conduct of providers to the public satisfaction with the system. The attitudes of the health personnel—particularly, nurses and doctors—were a major source of discontent for the participants. Some participants detailed instances of preferential treatment—whereby doctors attended to their acquaintances before others—during patient consultations:

*Some doctors skip Kofi to take care of Kojo because he knows him* (Interview 11)

One major issue identified was poor interpersonal skills, especially among the nurses. Some participants recounted instances of how some health personnel verbally abused them, while others shared situations of lack of empathy from the personnel:

*… Yes [*response to whether she had been verbally abused]*, where I went to give birth. The nurses shouted and insulted me, and the way they talk isn’t acceptable.* (Interview 6)

*These doctors will never comfort you; they just shout and ask you what is wrong with you and just ask you to go for the drug…. They should go back to school… I even curse them after visiting the hospital that they won’t live for long* (Interview 18)

Participants surmised that some of these poor attitudes resulted from the health personnel’s insufficient concern for their primary duties and their patients. They contended that health personnel showed greater concern for their private affairs while they were on duty. Some nurses, for instance, were sighted chatting on social media instead of caring for patients:

*… There is a lady who was very sick and went to the hospital; the doctor told her he had to close and so did not take care of her. … She went somewhere else* (Interview 5)

*My friend got sick and was taken to hospital … there were about six nurses, … they were all ‘WhatsApping’ [chatting via the WhatsApp platform]. They ignored us when we were talking to them … I was very angry, I fought with them* (Interview 36)

Indeed, one participant who was a nurse confirmed the prevalence of such poor professionalism among her peers:

*I had my clinical* [nursing practicum] *at one hospital, and because of the poor attitudes of some of my colleagues, I didn’t want to go there when I was posted.* (Interview 10)

Participants attributed such poor attitudes to mainly two factors, which were related to perceived weaknesses in institutional /organisational arrangements in the management of health facilities and personnel. The first was inadequate supervision in the health system (health facilities):

*The leaders in the hospitals don’t do their jobs well. ...they don’t monitor the nurses.* (Interview 21)

The second factor was questionable training. Participants held the view that some health personnel behave poorly due to their low commitment to the profession because they are ‘protocol nurses’—implying that such nurses gained admission to training schools because of their acquaintances with influential people. Others also claimed that many nurses were only in the profession for pecuniary reasons due to limited employment opportunities in the country:

*Some of them don’t qualify to become nurses. … Some even find it difficult to write their names* (Interview 9)

*I think many people have turned to the nursing profession because of money and unemployment. It’s easy to get a job as a nurse* (Interview 10)

To demonstrate their disdain for the poor attitudes of the health personnel, some participants had decided not to visit health facilities where the staff were discourteous even if it meant that they had to travel long distances to access needed care:

*The way they (nurses) shout at you, I’ve stopped visiting some hospitals. … I won’t even go there if it is closer to me* (Interview 5)

### Financial barriers to healthcare

The extent of satisfaction with the health system also had to do with financial challenges in accessing needed health services. Such challenges were held at *intrapersonal and public policy* levels, as some participants associated their satisfaction with the healthcare system to their economic situations and the weaknesses in public interventions meant to alleviate financial barriers to care. Some participants ascribed that the quality of care given to patients is proportionate to their financial status: the wealthier a patient is, the better is the care he or she receives and vice-versa. To many of them, the situation has become worse over time:

*… Previously, if you don’t have money and go to a hospital, they’ll take care of you, but now if you don’t have money and you go to the hospital, they’ll only give you a few medications (*Interview 12)

While health policy initiatives such as the NHIS in Ghana is meant to address financial barriers to healthcare, participants felt that its effect was minimal as patients still have to spend a significant amount on care:

*The government has told us to subscribe to health insurance (NHIS), but you won’t get the best of drugs if you go to the hospital with the NHIS* (Interview 11)

*You will get only paracetamol if you go with NHIS... For blood transfusion and quality drugs, you’ve got to buy them… So, you will die if you can’t afford them* (Interview 25)

Financial challenges, therefore, underlined the discontent of participants when it comes to health services in Ghana.

### Poor and inadequate health facilities and equipment

This theme also echoed the importance of community characteristics and organisation to public satisfaction, in addition to public policy aspects of the healthcare system. Some participants felt that there was too much pressure on existing health facilities, which has led to underperformance in many areas:

*... Our health system is very poor. … Korle-bu and KATH* [Two leading teaching hospitals] *were built a long time ago, but let’s ask which of the other regions have big hospitals like these ones? So, it puts pressure on all the big hospitals* (Interview 6)

Closely related to the issue of pressure on health facilities were experiences relating to inadequate and sometimes lack of health services in some rural communities. This meant that people in rural areas had to travel long distances to access basic healthcare:

*Around this village and the neighbouring communities, the closest clinic is at [name withheld] … If someone falls critically ill, they even die before we get them to the hospital* (Interview 27)

Moreover, the participants felt that most health facilities lacked essential equipment and facilities such as beds. Some recounted instances they had seen on the news to substantiate their assertions. They cited instances where patients were treated on the floor:

*One person went to three hospitals with all of them telling him that there were no beds, and he died* (Interview 6)

*Some patients were receiving treatment on the floor at Korle-bu. It was shown on GH-One TV… another person was receiving treatment in a taxi. Do we receive treatment in cars?* (Interview 9)

Others also complained that some health facilities were the cause of ill-health as they are unhygienic, filthy, and riddled with mosquitoes:

*Our hospitals are filthy. Nothing works at those places* (Interview 9)

*There are a lot of mosquitoes at some hospitals. If you don’t take care, you’ll get sick* while *you are there* (Interview 18)

### Perceived efficacy of services and care

The participants’ experiences also revealed how the organisation of the healthcare system, especially in clinics and hospitals, affects satisfaction with the healthcare system. Primarily their concern was the low efficacy of the health services. Their rating of the healthcare system stemmed from personal experiences and expectations as well as the experiences of their acquaintances. A major issue raised had to do with waiting times in hospitals and for other services such as ambulances:

*I was sick and went to the hospital. If it weren’t for God’s intervention, I would have died… I arrived at the hospital at 7 am, and the doctor came to me at 4 pm* (Interview 18)

*Ghana ambulance service won’t mind you even if you dial their number* (Interview 5)

Owing to these perceived inefficiencies [in the orthodox services described above], some participants revealed that they relied mainly on traditional medicine [usually unapproved and homemade herbal remedies]. This demonstrated their dissatisfaction with the public health system, which is dominated by orthodox medical practice. The dislike for orthodox medicine in favour of traditional medicine was often a result of perceived inefficacy of orthodox medical treatment:

*The last time I took an orthodox drug, it brought me sickness …. I took some blood tonic, and it caused me to have a hernia, so I turned to a herbal drug, and the hernia has not shown itself after 15 years* (Interview 14)

*The orthodox drugs kill people sometimes. … My brother died because of taking an injection* (Interview 18)

## Discussion

This study adopted a social ecological perspective to analyse public satisfaction with Ghana’s healthcare system. Even though there were variations in our participants’ experiences and the degree of satisfaction with the health system, the general impression was that participants were not entirely satisfied, which is consistent with earlier research [13]. Our qualitative and quantitative data findings show that factors influencing satisfaction with health systems cut across micro and macro levels, consistent with the current body of research [16, 28, 36].

### Intrapersonal domain and public satisfaction with the healthcare system

We found evidence that personal level factors and conditions significantly affect public satisfaction with the health system. Intrapersonal factors form the basis of behaviour, interest, and the needs people have in most situations [14, 41, 44]. They determine the extent to which existing and emerging conditions affect individuals [36]. These assertions were significantly substantiated in this study. It was found that older persons were more likely to be satisfied with the health system than younger persons. This finding is surprising given that dedicated health services for older persons in Ghana are considered poor [10, 39]. Older persons’ expectations about the health system are likely to be lower than those of younger people [59]. This is because older persons may have become accustomed to the poor nature of services to the extent that they do not recognise the weaknesses in the system [27]. We also observed that employed participants were more likely to be satisfied with the system than the unemployed, which correlates with existing literature [48]. People who are employed are likely to possess the financial wherewithal to pay for their healthcare expenses themselves, including travel expenses. Also, being economically engaged opens an opportunity to live an active lifestyle that contributes to positive health outcomes, which are known to be associated with satisfaction with the health system. Indeed, in this study, participants who perceived their health and well-being in positive terms were more likely to be satisfied with the health system as observed elsewhere [19, 27, 48]. Participants who described their health in favourable terms may have had positive outcomes after previous healthcare experience, which could spur satisfaction with the healthcare system [48]. People who are in poor health or unwell may be dissatisfied with the health system due to the poor or even complete absence of the essential services they need, as some of the participants in our qualitative study alluded. In addition, those with poor health are likely to use health services more regularly. Their repeated visits allow them to discover the limitations of the healthcare system and consequently cause dissatisfaction [59]. Given these findings, an objective of Ghana’s health system, going forward, must include strategies to ensure appropriate services and optimum outcomes for different categories of potential service users. For a start, the sociodemographic and disease profile of target populations must be continuously revisited in existing and new operational policies.

### Interpersonal domain of satisfaction with the healthcare system

We found in the quantitative study that people with sufficient linking SC were more likely to be satisfied with the healthcare system. In Ghana, linking SC sometimes includes relationships with professionals such as teachers, community and institutional leaders, and health professionals. People tend to use these social connections to seek health-related information and make decisions about health services [5, 11]. Therefore, sections of the public with adequate linking SC are more likely to be satisfied with the health system given the support they receive from their linking SC. However, according to our qualitative findings, linking SC and various forms of social networks can also be a source of dissatisfaction. The qualitative findings indicated that one attitude of health professionals that the public found distasteful was the special treatment of the professionals’ acquaintances. Indeed, much has been written about the ill-practices of health professionals in Ghana, and these findings offer insight into the consequences of such practices for the healthcare system [2, 29, 54].

Notwithstanding, our qualitative findings and extant literature indicate that the poor attitudes of the health personnel are not random individual behaviours but rather a reflection of prolonged weakness in organisational policies and strategies which have caused pressure on health facilities and services [15, 56]. Many health facilities in Ghana are under-resourced in terms of staff and essential equipment [56]. While the government and its partners have increased spending on the health sector, research shows that this expenditure was not based on need or rigorous equity-based principles [56]. Under-staffing and inadequate equipment lead to pressure on health personnel to perform beyond the limits of available resources, which can be a source of frustration leading to poor performance and, by extension, poor attitudes towards service users [17]. Despite the above assertions, our qualitative study also showed that the ratings and perception of satisfaction with the health system among some participants were based on their personal experiences and those of their social acquaintances. This speaks to the need to manage public satisfaction from an ecological point of view instead of a purely individual perspective because an individual’s ratings of satisfaction ‘are functions of social influence processes’ ([41], p. 580). Thus, the dynamics of social relationship formations and the functions of different kinds of social relationships within the sphere of influence of a given health system and its sub-sectors must be understood and incorporated into policymaking and every operation.

### Community domain and satisfaction with the healthcare system

The findings show that factors relating to the community domain of the social ecological model, such as area of residence, influenced satisfaction with the healthcare system. Urban residents were less likely to be satisfied with the system than rural residents. This is consistent with observations by earlier studies [27, 48, 64]. Urban residents are often more aware of their health needs and the ideal standards for health systems [3]. Such fundamental knowledge increases their expectations and the consequent likelihood of being disappointed if the available services are inefficient [40]. This is different in the case of rural residents, whose span of knowledge and expectations of the health system is limited and easier to satisfy [27]. With the advancement of the Community-Based Health Planning Scheme (CHPS) (see [5]), which promotes access to primary healthcare in rural areas in Ghana, this finding is not surprising and calls for more of these interventions in rural areas. Others argue that high public satisfaction in rural settings is associated with low pressure on facilities and services offered in these places [64]. However, this is unlikely the case in places like Ghana, where access to high-level medical services in rural areas is low [8, 56]. Because of this assertion, our finding implies that people living in rural areas, who are often disadvantaged in terms of health services provision [3, 44], are likely to be even more satisfied with the health system if further improvements can be made to services offered to them. In the context of the social-ecological perspectives on health, improving services to rural residents would require concerted policies to promote social and political integration within communities and externally to ensure that felt needs of residents are met under a common understanding of the broader sectoral policies [44, 53].

### Organisational and public policy aspects of public satisfaction with the healthcare system

Our findings indicate that public satisfaction is in one way or the other associated with factors related to characteristics of organisational and public policy domains. Participants who favoured more welfare policies and had an interest in politics, as well as those who trusted the health system, were more likely to be satisfied with the healthcare system than their respective counterparts, as also observed in related studies [27, 36]. From a purely social and public policy standpoint, these factors and their association with satisfaction with the health system are matters of self-interest (whether a person stands to benefit or be affected by a policy or situation) or ideological perspective (personal values and beliefs that affect attitudes) [36, 58]. For instance, in this study, it is likely that participants who were satisfied with the healthcare system were those who had benefited from policies such as the NHIS and LEAP programmes in one way or the other. Owusu-Addo et al. [52] observed that LEAP beneficiaries who are considered the poorest of the poor and by default given a waiver of the NHIS premium tend to have increased access to health services. Indeed, our qualitative findings point to financial challenges as a source of dissatisfaction among many participants. Existing studies indicate a general preference for more public welfare expenditure on healthcare [7]. This implies that more pragmatic measures must be implemented to ensure financial inclusivity in healthcare to improve satisfaction with the system. For one, the challenges with the NHIS must be addressed as a matter of urgency [32].

However, in the context of the social ecological model, these findings raise a critical question: how can the healthcare system cultivate trust and respond to the public’s political and welfare orientation about health provisions as part of its operational arrangements and policy design and implementation? This question is important because these factors are typically considered external to the health system [27]. The response to this question starts from a critical review of other domains of the social ecological model as presented in this paper. For instance, matters relating to the poor attitudes of health personal require a closer look at the organisational norms and values that engender positive relationships between the public and service providers. New and innovative approaches are required to re-orient health personnel and administrators towards their professional code of conduct. Nevertheless, such re-orientation can only be a short-term measure. One long term measure could be in the form of addressing fundamental challenges in personnel recruitment, training methodologies, and infrastructure gap, as pointed out by participants in the qualitative study. This measure will require cross-sectoral employment creation policies to offer alternative career opportunities and fair wages. In the long term, this can ensure that only those who are genuinely interested would venture into health-related professions, such as nursing. Such policies will ultimately improve public satisfaction with the health system. Another long-term policy at the macro level is a need for transparency in health policy formulation and implementation to make the public and key stakeholders feel part of the system. Such transparency will be an opportunity to understand their values, political and welfare policy interests, and perception about health care issues. This process will also engender trust between the public and the healthcare system and help to meet the service expectations of the public.

Dissatisfaction relating to the efficacy of the healthcare system also brings up the need to pay attention to important policy and service gaps in the health system. The preference that some participants have for informal healthcare calls for the intensification of efforts to integrate traditional medical practices into the formal services since traditional practices are still on the margins of public health services. While traditional medical practices are formally accepted in Ghana, their integration into the formal medical practice is undoubtedly weak [1, 56]. Thus, as was the case of some participants in this study, people preferring herbal and other traditional medical services would find it difficult to do so within the public healthcare sector, leading to dissatisfaction.

## Conclusions

To the best of our knowledge, this paper is the first to use the social ecological lens to examine public satisfaction with the healthcare system using a mixed-method design. We found evidence from quantitative and qualitative perspectives that factors relating to all four adapted domains of the social ecological model are associated with public satisfaction with healthcare. The quantitative aspect of the study provided evidence that intrapersonal factors (e.g., being older and having good health and well-being status); interpersonal factors (e.g., linking SC), community factors (e.g., living in rural areas), and organisation and public policy factors (e.g., trust in the health system, favouring welfare policies, and being interested in politics) are positively associated with satisfaction with the healthcare system. However, aspects of the qualitative study showed that some factors such as linking SC (i.e., interpersonal domain) are likely to have a negative effect on satisfaction with the healthcare system. Our interpretation of these findings shows that many factors associated with satisfaction with the health system are related to organisational and public policy domains of the social ecological model—i.e., prevailing healthcare and welfare policies and how the healthcare system is organised and operated.

From these findings, we argue that satisfaction with the healthcare system among members of the public is shaped by a multiplicity of factors, including the characteristics of individuals and their perception or conditions relating to their social and physical environments as well as policy arrangements. Hence, policy-driven analysis of healthcare system satisfaction should consider the multi-layered nature of the factors that inform the public’s perception. From our study, improving satisfaction with the healthcare system in places such as Ghana must incorporate precepts of the social-ecological model by considering the demographic profile, the health needs, political orientation, issues of trust in the healthcare system, and the dynamics and impact of social relationships of populations concerned.

### Limitations of the study

The findings of this study should be interpreted in light of some possible limitations. First, we do not seek to argue for causality as the study is based on cross-sectional data. This also means that readers must be cautious about generalising the findings beyond the context of this study. Second, the sample comprised the public and not patients, which means that the findings do not apply to patients or specific service users. While our findings cannot represent the actual experiences of service users, they provide a holistic perspective on the issue of public satisfaction with the healthcare system. Future studies can sample both the public and patients to gauge any potential differences in their perspectives. Third, as the dependent variable asked about general issues about the healthcare system, it is impossible to gauge which specific aspect (e.g., public health promotion, curative care services, preventive services) was the focus of participants in the survey. However, the qualitative findings of this study provide a perspective on where the interests of participants lie, which appears to be on curative healthcare. Fourth, our study focused mainly on perceptions relating to the public healthcare system. It thus cannot be said to provide a holistic perspective on satisfaction with the overall health system. To gain a broader perspective of satisfaction with the health system in Ghana, future satisfaction studies need to cover both public and private health facilities. Fifth, the regression model (Table 2) only explained about 17% of the variance in the dependent variable, indicating that other factors such as the use of private healthcare facilities excluded from the model can provide further explanation regarding satisfaction with the healthcare system. Finally, the instruments used to measure our dependent and independent variables, and the qualitative interviews, were primarily subjective. Therefore, there is a likelihood of social desirability bias, which may have influenced the views of participants who did not share their actual experiences. Future studies can adopt a more objective approach to ascertaining the public’s views on the healthcare system.

## Supplementary Information

**Additional file 1: Appendix I:** Interview guide; **Appendix II:** Sampling and data collection; **Appendix III:** Table 1: Spearman’s correlation analysis of variables in the study.

## Data Availability

The datasets analysed during the current study are available from the corresponding author. A request will be considered on a case-by-case basis.

## Abbreviations

CHPS: Community-Based Health Planning Scheme
CSIR: Council for Scientific and Industrial Research
GHS: Ghana Health Services
LEAP: Livelihood Empowerment against Poverty
MoH: Ministry of Health
NHIS: National Health Insurance Scheme
NHIA: National Health Insurance Authority
SC: Social capital
SES: Socioeconomic status
SPSS: Statistical Package for the Social Sciences
