# Supplementary material for: A multidimensional study of public satisfaction with the healthcare system: a mixed-method inquiry in Ghana
Source: BMC Health Serv Res. 2021 Dec 9;21:1320. doi: 10.1186/s12913-021-07288-1 (PMC8656047; doi:10.1186/s12913-021-07288-1)
Supplement: Supplementary file 1 — Additional file 1: Appendix I: Interview guide; Appendix II: Sampling and data collection; Appendix III: Table 1: Spearman’s correlation analysis of variables in the study. [file 12913_2021_7288_MOESM1_ESM.docx]

**A multidimensional study of public satisfaction with the healthcare system: a mixed-method inquiry in Ghana**

# Appendix I: Interview guide

(Below are the questions asked as part of the broader study relative to this article)

- Age,
- sex,
- educational level,
- place of residence (rural/urban),
- region of residence

1. What health services do you usually use? Why?
   1. Orthodox vs traditional
   2. Private vs public (focus mainly on public for detailed discussions)
   3. Primary healthcare (including pharmacies), secondary, tertiary facilities/services
2. Alternative treatments used aside from utilising formal (licensed health institutions)
   1. Which ones?
   2. Why?
3. In general, would you say that health services in Ghana are well-organised?
   1. Why?
4. Are you generally happy with health services in Ghana?
   1. Public services mainly (comparative discussions with private too)
   2. Why?
   3. Can you share some personal or household experiences regarding your happiness or otherwise with the health system?
5. What major factors put you off from accessing health services that you and your household sometimes need?
   1. Probe for issues of availability, affordability, geographical accessibility, accommodation of services, and acceptability of services reasons/experiences
6. What factors excite you about health services in Ghana?
   1. Why are these factors important to you?
7. If you had to change one thing about the health services in Ghana, what would that be?

# Appendix II: Sampling and data collection

*Quantitative study:*

We conveniently determined the number of districts in each region and the sample sizes for each region but with careful attention to the population sizes and the composition of rural and urban communities in each region. We used a systematic sampling technique to select one participant from every second house in rural areas and every fifth house in urban due to settlement sparsity and relatively small populations in rural areas as has been done elsewhere [1, 2].Using these approaches, we were able to recruit participants who adequately represented the country and regions of study in terms of age, sex, religion, socioeconomic conditions, geographic characteristics.

*Qualitative study*

In the qualitative study, we used semi-structured in-depth interviews to gather data from 39 participants who were interviewed mostly at their homes, and workplaces. The first and second authors conducted the interviews. While theoretical saturation was obtained after the 20^th^ interview, we carried out more interviews to provide a balance to the sample. We used the interpretivist paradigm to ensure that the experiences of participants remained at the centre of discussions and interpretation of data [3, 4]. Purposive sampling technique was used in all places to select participants to assure adequate representation of people in terms of age, sex, educational attainment, and rural-urban residence as shown in Table 3. The data were gathered from four regions including Ashanti, Brong Ahafo, Upper East, and Greater Accra regions with the help of research assistants who were fluent in the dominant native languages in the regions. For this paper, the discussions in each interview covered general and specific issues about how people felt about the health system, the challenges they faced in accessing health services, and the positive aspects of the system. Each interview lasted approximately 45 minutes, which were audio-recorded. All the researchers were familiar with the research context (in terms of the health system and prevailing socioeconomic conditions in the study area). However, the researchers had no prior knowledge of the participants. The interviews were transcribed in the language of interview and later translated into English by qualified persons. Field notes that were made during the data collection were added to the data for analyses. The transcripts were reviewed by an expert in social health research. Further details of the methods employed in the study have been reported elsewhere [5]

# Appendix III: Table 1: Spearman's correlation analysis of variables in the study

|  |  | 1 | 2 | 3 | 4 | 5 | 6 | 7 | 8 | 9 | 10 | 11 | 12 | 13 | 14 | 15 | 16 | 17 | 18 | 19 | 20 |
| --- | --- | --- | --- | --- | --- | --- | --- | --- | --- | --- | --- | --- | --- | --- | --- | --- | --- | --- | --- | --- | --- |
|  | Satisfaction with health system | 1.000 |  |  |  |  |  |  |  |  |  |  |  |  |  |  |  |  |  |  |  |
|  | Age | .135^**^ | 1.000 |  |  |  |  |  |  |  |  |  |  |  |  |  |  |  |  |  |  |
|  | Sex | -0.021 | 0.036 | 1.000 |  |  |  |  |  |  |  |  |  |  |  |  |  |  |  |  |  |
|  | Duration of stay | .077^**^ | .415^**^ | -0.020 | 1.000 |  |  |  |  |  |  |  |  |  |  |  |  |  |  |  |  |
|  | Religiosity | 0.016 | -.078^**^ | .084^**^ | -.079^**^ | 1.000 |  |  |  |  |  |  |  |  |  |  |  |  |  |  |  |
|  | Highest education | -0.034 | .147^**^ | -.057^*^ | 0.028 | 0.020 | 1.000 |  |  |  |  |  |  |  |  |  |  |  |  |  |  |
|  | Health status | .094^**^ | -.280^**^ | -.075^**^ | -.214^**^ | 0.037 | -0.038 | 1.000 |  |  |  |  |  |  |  |  |  |  |  |  |  |
|  | Happiness | .087^**^ | -.125^**^ | -0.006 | -.183^**^ | .126^**^ | 0.036 | .442^**^ | 1.000 |  |  |  |  |  |  |  |  |  |  |  |  |
|  | Rural/Urban | .080^**^ | .121^**^ | 0.019 | .277^**^ | -0.029 | -.078^**^ | -.151^**^ | -.072^**^ | 1.000 |  |  |  |  |  |  |  |  |  |  |  |
|  | Household size | 0.022 | .142^**^ | 0.013 | .288^**^ | -0.024 | 0.023 | -.136^**^ | -.097^**^ | .220^**^ | 1.000 |  |  |  |  |  |  |  |  |  |  |
|  | Employment status | -.090^**^ | -.104^**^ | 0.050 | .127^**^ | 0.013 | -0.027 | -.123^**^ | -.118^**^ | .055^*^ | .143^**^ | 1.000 |  |  |  |  |  |  |  |  |  |
|  | SES | 0.035 | 0.017 | -.059^*^ | 0.009 | -0.022 | -0.015 | .062^*^ | .082^**^ | 0.023 | 0.041 | -.083^**^ | 1.000 |  |  |  |  |  |  |  |  |
|  | Income/stipend | -0.011 | 0.055 | -.118^**^ | -.192^**^ | -0.061 | .193^**^ | .210^**^ | .082^*^ | -.214^**^ | -.166^**^ | -.301^**^ | .079^*^ | 1.000 |  |  |  |  |  |  |  |
|  | Region | 0.044 | .244^**^ | 0.009 | .389^**^ | -0.021 | 0.018 | -.188^**^ | -.125^**^ | .275^**^ | .386^**^ | .092^**^ | -.060^*^ | -.303^**^ | 1.000 |  |  |  |  |  |  |
|  | Welfare support | .080^**^ | 0.045 | 0.033 | 0.052 | .094^**^ | 0.003 | -0.052 | -0.018 | .116^**^ | 0.047 | -0.003 | -0.035 | -.113^**^ | .082^**^ | 1.000 |  |  |  |  |  |
|  | Bonding SC | 0.009 | 0.024 | -0.008 | .165^**^ | -.059^*^ | -0.012 | 0.035 | 0.045 | -0.004 | 0.047 | 0.042 | .062^*^ | .078^*^ | .083^**^ | -.091^**^ | 1.000 |  |  |  |  |
|  | Bridging SC | .062^*^ | .084^**^ | -0.044 | .117^**^ | -.131^**^ | -0.027 | 0.034 | -0.028 | -0.021 | 0.040 | -0.005 | 0.033 | .117^**^ | .121^**^ | -0.003 | .584^**^ | 1.000 |  |  |  |
|  | Linking SC | .100^**^ | .152^**^ | -0.035 | .146^**^ | -.140^**^ | -.074^**^ | .063^*^ | 0.036 | -.056^*^ | 0.052 | -.071^*^ | .066^*^ | .147^**^ | .137^**^ | -.064^*^ | .497^**^ | .748^**^ | 1.000 |  |  |
|  | Trust in health system | .247^**^ | .107^**^ | .066^*^ | .077^**^ | .110^**^ | .055^*^ | -0.032 | 0.035 | .071^**^ | 0.001 | -.063^*^ | -0.038 | -.096^*^ | .102^**^ | .075^**^ | .065^*^ | .061^*^ | .085^**^ | 1.000 |  |
|  | Interest in politics | .133^**^ | .112^**^ | -.097^**^ | .074^**^ | .108^**^ | 0.019 | .100^**^ | .156^**^ | -0.001 | 0.002 | -.120^**^ | 0.054 | 0.047 | 0.047 | 0.041 | .094^**^ | .077^**^ | .109^**^ | .090^**^ | 1.000 |

References

1. Amoah, P.A., *Social participation, health literacy, and health and well-being: A cross-sectional study in Ghana.* SSM - Population Health, 2018. **4**: p. 263-270.

2. Gyasi, R.M., D.R. Phillips, and P.A. Amoah, *Multidimensional Social Support and Health Services Utilization Among Noninstitutionalized Older Persons in Ghana.* Journal of Aging and Health, 2020: p. 0898264318816217.

3. Bryman, A., *Social Research Methods*. Fourth ed. 2012, Oxford: Oxford University Press.

4. Creswell, J.W., *Research Design: Qualitative, Quantitative and Mixed Methods Approaches*. Fourth Edition ed. 2014, London: Sage.

5. Amoah, P.A., *Examining Attitudes towards Welfare in an In/Security Regime: Evidence from Ghana.* Social Policy and Society, 2020. **19**(4): p. 1-16.
